# Peripheral Oxidative Stress Biomarkers in Spinocerebellar Ataxia Type 3/Machado–Joseph Disease

**DOI:** 10.3389/fneur.2017.00485

**Published:** 2017-09-20

**Authors:** Adriano M. de Assis, Jonas Alex Morales Saute, Aline Longoni, Clarissa Branco Haas, Vitor Rocco Torrez, Andressa Wigner Brochier, Gabriele Nunes Souza, Gabriel Vasata Furtado, Tailise Conte Gheno, Aline Russo, Thais Lampert Monte, Raphael Machado Castilhos, Artur Schumacher-Schuh, Rui D’Avila, Karina Carvalho Donis, Carlos Roberto de Mello Rieder, Diogo Onofre Souza, Suzi Camey, Vanessa Bielefeldt Leotti, Laura Bannach Jardim, Luis Valmor Portela

**Affiliations:** ^1^Programa de Pós-Graduação em Ciências Biológicas: Bioquímica, Universidade Federal do Rio Grande do Sul (UFRGS), Porto Alegre, Brazil; ^2^Programa de Pós-Graduação em Saúde e Comportamento, Centro de Ciências da Vida e da Saúde, Universidade Católica de Pelotas (UCPel), Pelotas, Brazil; ^3^Programa de Pós-Graduação em Medicina: Ciências Médicas, Universidade Federal do Rio Grande do Sul (UFRGS), Porto Alegre, Brazil; ^4^Serviço de Genética Médica, Hospital de Clínicas de Porto Alegre (HCPA), Porto Alegre, Brazil; ^5^Serviço de Neurologia, Hospital de Clínicas de Porto Alegre (HCPA), Porto Alegre, Brazil; ^6^Laboratório de Identificação Genética, Hospital de Clínicas de Porto Alegre (HCPA), Porto Alegre, Brazil; ^7^Departamento de Medicina Interna, Universidade Federal do Rio Grande do Sul (UFRGS), Porto Alegre, Brazil; ^8^Programa de Pós-Graduação em Genética e Biologia Molecular, Universidade Federal do Rio Grande do Sul (UFRGS), Porto Alegre, Brazil; ^9^Departamento de Neurologia, Universidade Federal de Ciências da Saúde de Porto Alegre (UFCSPA), Porto Alegre, Brazil; ^10^Departamento de Bioquímica, Instituto de Ciências Básicas da Saúde (ICBS), Universidade Federal do Rio Grande do Sul (UFRGS), Porto Alegre, Brazil; ^11^Programa de Pós-Graduação em Epidemiologia, Universidade Federal do Rio Grande do Sul (UFRGS), Porto Alegre, Brazil; ^12^Departamento de Estatística, Universidade Federal do Rio Grande do Sul (UFRGS), Porto Alegre, Brazil

**Keywords:** spinocerebellar ataxia type 3, Machado–Joseph disease, oxidative stress, reactive oxygen species, polyglutamine disorders

## Abstract

**Objectives:**

Spinocerebellar ataxia type 3/Machado–Joseph disease (SCA3/MJD) is a polyglutamine disorder with no current disease-modifying treatment. Conformational changes in mutant ataxin-3 trigger different pathogenic cascades, including reactive oxygen species (ROS) generation; however, the clinical relevance of oxidative stress elements as peripheral biomarkers of SCA3/MJD remains unknown. We aimed to evaluate ROS production and antioxidant defense capacity in symptomatic and presymptomatic SCA3/MJD individuals and correlate these markers with clinical and molecular data with the goal of assessing their properties as disease biomarkers.

**Methods:**

Molecularly confirmed SCA3/MJD carriers and controls were included in an exploratory case–control study. Serum ROS, measured by 2′,7′-dichlorofluorescein diacetate (DCFH-DA) as well as superoxide dismutase (SOD) and glutathione peroxidase (GSH-Px) antioxidant enzyme activities, levels were assessed.

**Results:**

Fifty-eight early/moderate stage symptomatic SCA3/MJD, 12 presymptomatic SCA3/MJD, and 47 control individuals were assessed. The DCFH-DA levels in the symptomatic group were 152.82 nmol/mg of protein [95% confidence interval (CI), 82.57–223.08, *p* < 0.001] higher than in the control and 243.80 nmol/mg of protein (95% CI, 130.64–356.96, *p* < 0.001) higher than in the presymptomatic group. The SOD activity in the symptomatic group was 3 U/mg of protein (95% CI, 0.015–6.00, *p* = 0.048) lower than in the presymptomatic group. The GSH-Px activity in the symptomatic group was 13.96 U/mg of protein (95% CI, 5.90–22.03, *p* < 0.001) lower than in the control group and 20.52 U/mg of protein (95% CI, 6.79–34.24, *p* < 0.001) lower than in the presymptomatic group and was inversely correlated with the neurological examination score for spinocerebellar ataxias (*R* = −0.309, *p* = 0.049).

**Conclusion:**

Early/moderate stage SCA3/MJD patients presented a decreased antioxidant capacity and increased ROS generation. GSH-Px activity was the most promising oxidative stress disease biomarker in SCA3/MJD. Further longitudinal studies are necessary to identify both the roles of redox parameters in SCA3/MJD pathophysiology and as surrogate outcomes for clinical trials.

## Introduction

Machado–Joseph disease (MJD), also referred to as spinocerebellar ataxia type 3 (SCA3/MJD), is an autosomal dominant neurodegenerative disorder caused by a CAG repeat expansion (CAGexp) at *ATXN3*, the gene that codes for ataxin-3. SCA3/MJD is part of the so-called group of polyglutamine (PolyQ) disorders (1); it is the most common form of SCA worldwide (2), with a minimal prevalence of 6:100,000 in Rio Grande do Sul, Brazil (3, 4).

Spinocerebellar ataxia type 3/Machado–Joseph disease onset typically occurs at approximately 32–40 years of age (2, 3, 5, 6). Gait ataxia is the main neurological sign; however, ataxia subsequently affects speech, swallowing, and limb coordination. Pyramidal, extrapyramidal, and peripheral nerve findings also occur during the disease course (1). The median survival time after onset is 21 years (7).

Expansion of the polyQ tract induces conformational changes in ataxin-3, which affects many properties of the protein, including stability and degradation (8), subcellular localization (9), molecular interactions with other proteins (10–12), and propensity to aggregate (9).

Aggregates of mutant polyQ proteins may be taken up by neuronal mitochondria, which leads to disturbances in the membrane potential and a subsequent increase in reactive oxygen species (ROS) production (13). Recent studies have demonstrated an imbalance between the ROS production and antioxidant defense capacity in patients and cellular models of SCA3/MJD, linking these abnormalities to the neurodegenerative process of the disease (12–15). In addition, oxidative stress increases ataxin-3 nuclear localization (16), a crucial step for SCA3/MJD *in vivo* phenotypic manifestation (17).

Natural history (NH) studies with well-validated SCA scales have indicated a very slow progression of ataxic and non-ataxic signs in SCA3/MJD (18–20) and have stressed the need for large sample sizes to test disease-modifying therapies in future randomized clinical trials (RCTs) (18–24). Surrogate biomarkers may hasten RCT and drug discoveries for SCA3/MJD, a disorder with no modifying treatment to date. The potential clinical relevance of oxidative stress elements as peripheral biomarkers of SCA3/MJD in patients has rarely been addressed (12, 15).

Considering the hypothesis of an oxidative stress imbalance in SCA3/MJD that may precede disease onset, we aimed to evaluate the peripheral ROS production and antioxidant defense capacity in symptomatic and presymptomatic SCA3/MJD individuals and a control population. We also evaluated the relationship of these markers with clinical and molecular data, with the goal to assess their properties as disease biomarkers.

## Materials and Methods

### Population, Design, and Eligibility Criteria

Symptomatic patients with a molecular diagnosis of SCA3/MJD or asymptomatic individuals with a normal neurological examination at 50% risk of SCA3/MJD on the basis of an affected first-degree relative who were seeking presymptomatic testing were recruited for this single-site, exploratory, case–control study at the Neurogenetics outpatients clinic, Hospital de Clinicas de Porto Alegre, from May 2011 to July 2013.

The data for symptomatic patients were collected during the baseline assessments of a RCT, in which a disease duration of more than 10 years and an inability to walk independently (canes, sticks, or walkers were allowed) were exclusion criteria (23). The control group consisted of previously at-risk individuals who did not carry CAG_exp_ at *ATXN3* plus healthy unrelated individuals with age, gender, and environmental characteristics similar to symptomatic individuals. Thyroid, renal, or hepatic disorders or a history of other significant neurological or systemic medical disorders were also exclusion criteria.

### Molecular and Clinical Evaluations

The *ATXN3* expanded region was analyzed as previously described (25). The Neurological Examination Score for Spinocerebellar Ataxias (NESSCA) (26) and the Scale for the Assessment and Rating of Ataxia (SARA) (27) were performed in all symptomatic individuals. The disease duration and age at disease onset of first symptom were provided by the patients and/or relatives. The median estimated age at onset of the presymptomatic group was calculated with the individuals’ current age and CAG_exp_ length in *ATXN3*, as previously reported (28).

### Sample Collection

Biological material collection was performed between 8 a.m. and 4 p.m. under fasting conditions. Serum was obtained *via* blood centrifugation at 6,000 × *g* for 5 min, frozen immediately, and stored at −80°C until analysis.

### Redox Assays

All samples and standards were measured in triplicate with variation coefficients <10%.

#### ROS Levels

To assess the ROS levels, 2′,7′-dichlorofluorescein diacetate (DCFH-DA) (Sigma-Aldrich, St. Louis, MO, USA) was used as a probe (29). An aliquot of the serum sample (60 µl) was incubated with DCFH-DA (final concentration 100 µM) at 37°C, in the dark, for 30 min. DCFH oxidation was measured fluorimetrically, using 488 nm excitation and 525 nm emission wave lengths. A standard curve using standard DCF (Sigma-Aldrich, St. Louis, MO, USA) (0.25–10 mM) was performed in parallel with the samples, and the results were expressed as nanomoles per milligram protein.

#### Antioxidant Enzyme Activities

The superoxide dismutase (SOD) (EC 1.15.1.1) activity was assessed by quantifying the inhibition of superoxide-dependent auto-oxidation of epinephrine and analyzing the absorbance of the samples at 480 nm. In microplate wells that contained serum samples (30 µl–60 µg of protein), 140 µl of glycine buffer (final concentration 50 mM; pH 10.2) and 10 µl of catalase (Sigma-Aldrich, St. Louis, MO, USA) (EC 1.11.1.6) (final concentration 10 µM) were added. In standard wells, only 180 µl of glycine buffer (final concentration 50 mM; pH 10.2) and 10 µl of catalase were added (final concentration 10 µM). The reaction was initiated by the addition of 10 µl of epinephrine (Sigma-Aldrich, St. Louis, MO, USA) (final concentration 60 mM) in all wells. The zero time absorbance was obtained at 480 nm, followed by recording the absorbance after 10 min at 32°C. The SOD activity unit was defined as the required enzyme amount to inhibit epinephrine oxidation by 50%. Data were expressed as units per milligram protein (29).

The glutathione peroxidase (GSH-Px, EC 1.11.1.9) activity was measured in a 96-well plate according to the method described by Wendel (30) using tert-butyl hydroperoxide as the substrate (Sigma-Aldrich, St. Louis, MO, USA). Nicotinamide adenine dinucleotide phosphate (NADPH) disappearance was monitored spectrophotometrically at 340 nm in a medium that contained (final concentrations): 2 mM of reduced glutathione (GSH), 0.15 U/ml glutathione reductase (GR) (EC 1.8.1.7), 0.4 mM azide, 0.5 mM tert-butyl hydroperoxide, and 0.1 mM NADPH plus serum sample (40 µl–80 µg of protein). One GSH-Px unit was defined as 1 µmol of NADPH consumed per minute, and the specific activity is represented as units per milligram of protein.

### Statistical Analysis

All investigated variables showed a normal distribution *via* a one-sample Kolmogorov–Smirnov test. The baseline characteristics among the groups were compared with ANOVA, independent samples *t*-test or chi-square test. The ROS levels and antioxidant enzyme activities among the groups were compared using the Generalized Linear Model (GLM) corrected for age. Differences between the specific groups in the GLM were assessed using Wald chi-square with Bonferroni correction for multiple comparisons. Correlations were performed with the Pearson correlation test, followed by a linear regression model when required. Statistical significance was defined as *p* < 0.05.

The *Ζ*-score was calculated using the following formula:

$$Z=\frac{x-\text{μ}}{\text{σ}}$$

where *x* is the sample value (raw score), μ is the population mean, and σ is the standard deviation of the population. The absolute value of *Ζ* represents the distance between the raw score and the population mean in units of SD (31).

### Ethics

The study was approved by the Ethics in Research Committee of our institution (register number 10-513). Written informed consent was obtained from all subjects prior to participation.

## Results

Fifty-eight individuals comprised the early/moderate stage symptomatic SCA3/MJD group, 12 individuals comprised the asymptomatic SCA3/MJD group, and 47 individuals comprised the healthy control group (9 related and 38 unrelated to the SCA3/MJD individuals). The clinical and demographic characteristics are described in Table 1.

**Table 1:** Demographics of the enrolled individuals.

	Healthy controls	Symptomatic SCA3/MJD	Presymptomatic	
	Mean (SD)	Mean (SD)	Mean (SD)	*p*
*N*	47	58	12	
Age (years)	40.7 (13.3)	40.6 (9.6)	32.4 (8.1)	0.053^a^
Female sex (*n*, %)	27 (57.4)	29 (48.3)	7 (58.3)	0.596^b^
Age at onset (years)	–	34.5 (9.1)	–	
Disease duration (years)	–	5.3 (2.5)	–	
CAG_exp_ length	–	75.4 (3.2)	73.5 (3.0)	0.071^c^
NESSCA	–	14.2 (4.8)	–	
SARA	–	9.7 (4.1)	–	
Predicted disease onset (years)	–	–	5.5 (2.1)	

*^a^ANOVA*.

*^b^Chi-square*.

*^c^Student’s t-test*.

*NESSCA, Neurological Examination Score for Spinocerebellar Ataxias; SARA, Scale for the Assessment and Rating of Ataxia; SCA3/MJD, spinocerebellar ataxia type 3/Machado–Joseph disease*.

**Figure 1:**
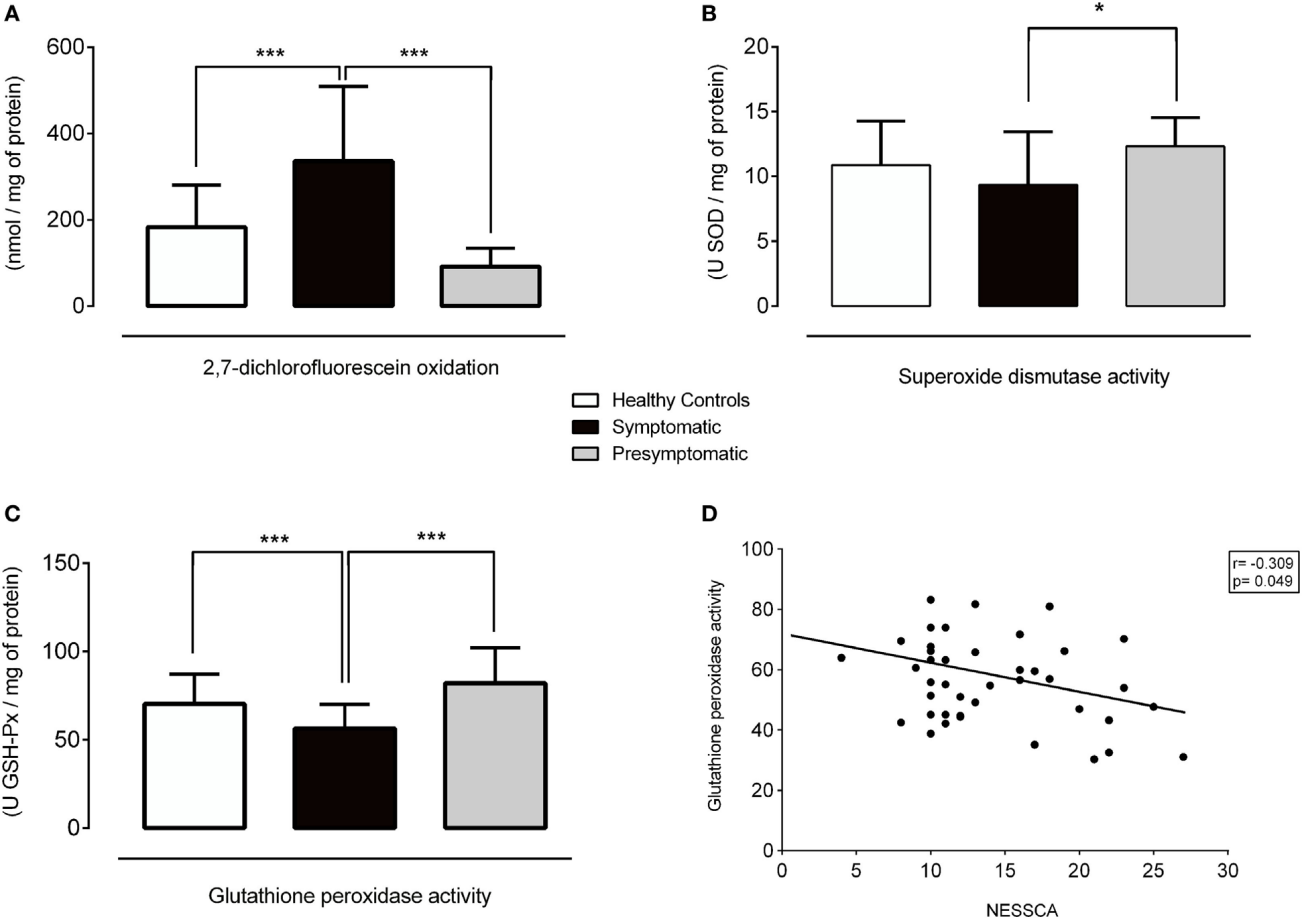
Redox parameters in healthy controls, symptomatic, and presymptomatic SCA3/MJD individuals. **(A)** ROS levels, **(B)** SOD activity, **(C)** GSH-Px activity and **(D)** Correlation between GSH-Px and NESSCA. Values are presented as the means, and error bars represent standard error; *p < 0.05 and ***p < 0.001. GSH-Px, glutathione peroxidase; NESSCA, Neurological Examination Score for Spinocerebellar Ataxias; ROS, reactive oxygen species; SCA3/MJD, spinocerebellar ataxia type 3/Machado–Joseph disease; SOD, superoxide dismutase.

**Figure 2:**
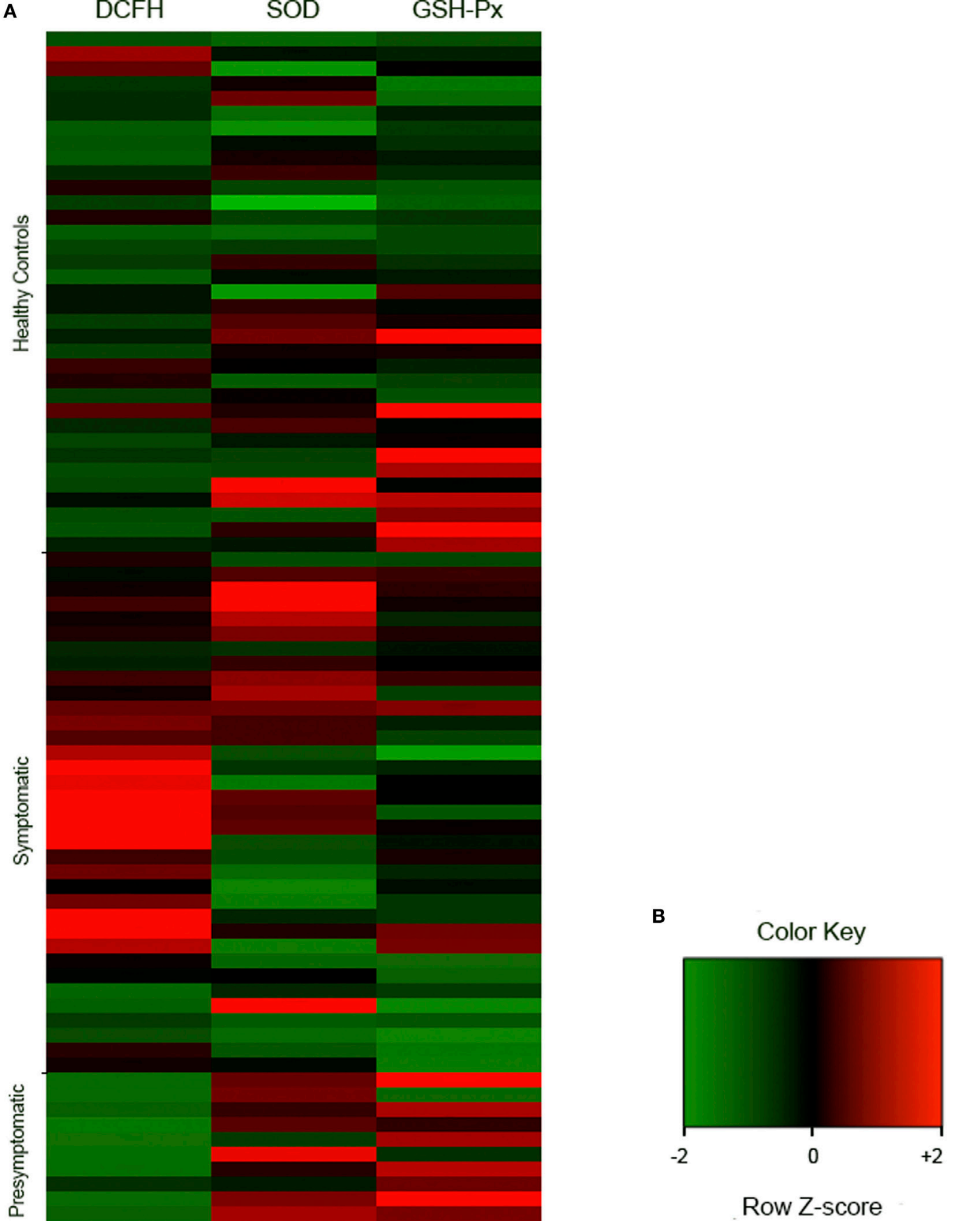
Representative cluster of the redox analyses in healthy controls (*n* = 35), symptomatic (*n* = 35), and presymptomatic (*n* = 10) SCA3/MJD individuals. The results were analyzed as *Ζ*-score values. *Ζ* is negative when the sample value is below the population mean and positive when it is above the mean. **(A)** Each square represents one individual. DCFH-DA, 2′,7′-dichlorofluorescein diacetate; GSH-Px, glutathione peroxidase; SCA3/MJD, spinocerebellar ataxia type 3/Machado–Joseph disease; SOD, superoxide dismutase. **(B)** Color key of row *z*-score.

The serum DCFH-DA levels (*p* < 0.001) and the SOD (*p* = 0.026) and GSH-Px (*p* < 0.001) activities were different among some of the groups (Figures 1 and 2, Table S1 in Supplementary Material).

The levels of the ROS marker DCFH-DA in the symptomatic group were 152.82 nmol/mg of protein [95% confidence interval (CI), 82.57–223.08, *p* < 0.001] higher than in the control and 243.80 nmol/mg of protein (95% CI, 130.64–356.96, *p* < 0.001) higher than in the presymptomatic group. The DCFH-DA levels were similar between the presymptomatic SCA3/MJD and control individuals (90.97 nmol/mg of protein, 95% CI, −21.83 to 203.79, *p* = 0.161). Refer to Figures 1A and 2A.

The SOD activity was 3 U SOD/mg of protein (95% CI, 0.015–6.00, *p* = 0.048) lower in the symptomatic than that of the presymptomatic SCA3/MJD group. There were no differences in the SOD activity between the symptomatic SCA3/MJD and control groups (1.51 U SOD/mg of protein, 95% CI, −0.37 to 3.39, *p* = 0.165) or the presymptomatic SCA3/MJD and control groups (1.49 U SOD/mg of protein, 95% CI, −1.51 to 4.50, *p* = 0.700). Refer to Figures 1B and 2A.

The GSH-Px activity in the symptomatic SCA3/MJD was 13.96 U GSH-Px/mg of protein (95% CI, 5.90–22.03, *p* < 0.001) lower than in the control and 20.52 U GSH-Px/mg of protein (95% CI, 6.79–34.24, *p* < 0.001) lower than in the presymptomatic SCA3/MJD group. The GSH-Px activity was similar between the presymptomatic SCA3/MJD and control groups (6.55 U GSH-Px/mg of protein, 95% CI, −7.19 to 20.30, *p* = 0.762). Refer to Figures 1C and 2A.

### Oxidative Stress Parameter Correlations with Clinical and Molecular Findings

The DCFH-DA levels and SOD activity did not correlate with the clinical features or molecular data in the symptomatic group (Figure S1 in Supplementary Material). The GSH-Px activity exhibited an inverse correlation with the NESSCA severity in the symptomatic group (*R* = −0.309, *p* = 0.049, Figure 1D), i.e., lower levels of GSP-Px activity were identified in the patients with more severe disease. No significant correlations of oxidative stress markers with predicted age of onset or CAG expansion length were identified in the presymptomatic SCA3/MJD group (*p* > 0.05 for all comparisons, Figure S2 in Supplementary Material).

## Discussion

This study evaluated redox parameters in a representative sample of symptomatic SCA3/MJD patients and presymptomatic carriers. The ROS marker DCFH-DA was higher in the symptomatic SCA3/MJD individuals; the activity of the antioxidant enzyme GSH-Px was lower in the symptomatic SCA3/MJD group and was associated with the disease severity; the presymptomatic carriers presented a higher activity of the antioxidant enzyme SOD than the symptomatic SCA3/MJD patients. Overall, these results indicate a pro-oxidative stress state in the early/moderate stages of SCA3/MJD and a potential antioxidant defense response in presymptomatic carriers.

In the present study, we identified higher ROS in the symptomatic SCA3/MJD patients measured by the DCFH-DA serum levels. The lack of correlations between the DCFH-DA levels and clinical or molecular data should be considered in the perspective of a peripheral marker for a disease that mainly affects the CNS. Nevertheless, our results differed from a clearly underpowered case–control study (seven SCA3/MJD and seven control individuals) that reported no differences in the DCFH-DA levels between groups (15).

Oxidative stress damage has been involved in several pathological processes that affect various organs and tissues. Specifically, the brain is particularly vulnerable as denoted by many neurodegenerative diseases of which oxidative stress has been implicated in the pathogenesis (16, 32). As previously stated, neuronal mitochondria function may be directly impaired by polyQ aggregates, which may lead to increased ROS generation (13). Alternatively or simultaneously, ROS may increase in SCA3/MJD as a result of a direct failure of antioxidant enzymes.

In our study, we identified higher SOD activity in the presymptomatic than early/moderate stages of symptomatic SCA3/MJD individuals. Furthermore, the GSH-Px activity was lower in the symptomatic SCA3/MJD group, and these lower levels were correlated with greater disease severity as measured *via* the ataxia and non-ataxia sign scale NESSCA. Pacheco et al. (15) identified similar results, in which the thiol levels, an indirect measure of GSH-Px activity, were lower in SCA3/MJD patient sera. Therefore, the GSH-Px activity was the most promising oxidative stress disease biomarker in SCA3/MJD, and our results strengthened the findings of previous indirect studies on the GSH-Px activity and present novel data that correlated this marker with disease severity.

According to our data, significant oxidative stress was only present after disease onset, and we speculate whether a failure/exhaustion in antioxidant defense mechanisms (depicted by an increased activity of SOD in the presymptomatic stages) may play a role in disease onset. Higher peripheral SOD activity in presymptomatic individuals than controls was a remarkable finding that departs from the previous scenarios. The hypothesis of an antioxidant defense response present in presymptomatic carriers is appealing, particularly in face of preliminary evidence that suggests alterations in other pathways are present in the preclinical phases of this disease (33). Nevertheless, it remains necessary to determine whether high SOD activity in the prodromal phases represents a phenomenon common to other tissues, such as the CNS. However, we emphasize that experimental evidence indicates a link between oxidative stress and SCA3/MJD pathology. The brain has a cellular defense system against oxidative stress, which includes high levels of several antioxidant enzymes, including SOD, GSH-Px, GSH reductase, and catalase (14). GSH plays predominant roles in the removal of excess H_2_O_2_ from the brain (14), as well as SOD in the removal of ${\text{O}}_{2}^{-}$ (32). Lower GSH levels and decreased activity of GSH reductase, catalase, and SOD were previously identified in mutant SCA3/MJD cell lines compared with wild-type cells, which suggests that mutant ataxin-3 may influence the activity of enzymatic components to efficiently remove both ${\text{O}}_{2}^{-}$ and H_2_O_2_ (14). Ataxin-3 binds to target gene promoters and modulates transcription by interacting with transcriptional regulators. During oxidative stress, ataxin-3 and forkhead box O (FOXO) transcription factor FOXO4 translocate to the nucleus, concomitantly bind to the SOD2 gene promoter and increase the expression of the antioxidant enzyme SOD2. Mutant ataxin-3 has a reduced capability to activate FOXO4-mediated SOD2 expression (12). The downregulation of SOD2 was confirmed in pons tissue and lymphoblastoid cell lines of SCA3/MJD patients. In SCA3/MJD patient lymphoblastoid cell lines, an impairment to upregulate SOD2 expression in association with a significant increase in ROS formation and cytotoxicity were reported, which suggests that a decreased antioxidant capacity and increased susceptibility toward oxidative stress contribute to neuronal cell death (12).

The general characteristics of the recruited sample were similar to the local SCA3/MJD population (4), such as the gender proportion, age at onset, and range of CAGexp. Despite several strengths, our study has several limitations. As we designed an exploratory case–control study, no primary outcome was elected, and no sample size calculation or study power determinations were performed. We conducted an exploratory study because of the lack of data on redox markers in SCA3/MJD patients and we would not have available the number of presymptomatic individuals necessary for an adequately powered study. As our sample sizes are one of the largest on biomarker studies of this disorder, the number of recruited individuals represents one of the study’s merits.

In conclusion, early/moderate stage symptomatic SCA3/MJD patients presented a similar peripheral redox profile to the previously reported SCA3/MJD cellular models: a decreased antioxidant capacity and an increased production of ROS. Additional collaborative studies with larger sample sizes of presymptomatic individuals and a prospective design may help to uncover the NH of these markers and their roles as surrogate (disease or treatment) biomarkers for future clinical trials, as well as understand the association between the failure of the antioxidant defense and disease onset and its potential therapeutic implications.

## Ethics Statement

The study was approved by the Ethics in Research Committee of our institution (register number 10-513). Informed written consent was obtained from all subjects prior participation.

## Author Contributions

AM and JA were responsible for the design, acquisition, analysis, interpretation, drafting, and approval of the final version of the manuscript. AL, CH, VRT, AB, GS, GF, TG, AR, TM, RC, AS, RD, KD, SC, and VBL were responsible for acquisition, analysis, interpretation, and approval of the final version of the manuscript. DS and CM were responsible for interpretation, drafting, critical revision, and approval of the final version of the manuscript. LJ and LP were responsible for the design, interpretation, drafting, critical revision, and approval of the final version of the manuscript.

## Conflict of Interest Statement

The authors declare that the research was conducted in the absence of any commercial or financial relationships that could be construed as a potential conflict of interest.

## Supplementary Material

Figure S1 Correlations of redox parameters with clinical and molecular data in the symptomatic SCA3/MJD group. **p* < 0.05. DCFH, 2′,7′-dichlorofluorescein diacetate; GSH-Px, glutathione peroxidase; NESSCA, Neurological Examination Score for Spinocerebellar Ataxias; SARA, Scale for the Assessment and Rating of Ataxia; SCA3/MJD, spinocerebellar ataxia type 3/Machado–Joseph disease; SOD, superoxide dismutase.

Figure S2 Correlations of redox parameters with clinical and molecular data in the presymptomatic SCA3/MJD group. Predicted time to onset (in years) was calculated with the formula: current age—predicted age of onset [according to Ref. (28)]. DCFH, 2′,7′-dichlorofluorescein diacetate; GSH-Px, glutathione peroxidase; SCA3/MJD, spinocerebellar ataxia type 3/Machado–Joseph disease; SOD, superoxide dismutase.
