# Supplementary figures and images for: Peripheral Oxidative Stress Biomarkers in Spinocerebellar Ataxia Type 3/Machado–Joseph Disease
Source: Front Neurol. 2017 Sep 20;8:485. doi: 10.3389/fneur.2017.00485 (PMC5611390; doi:10.3389/fneur.2017.00485)

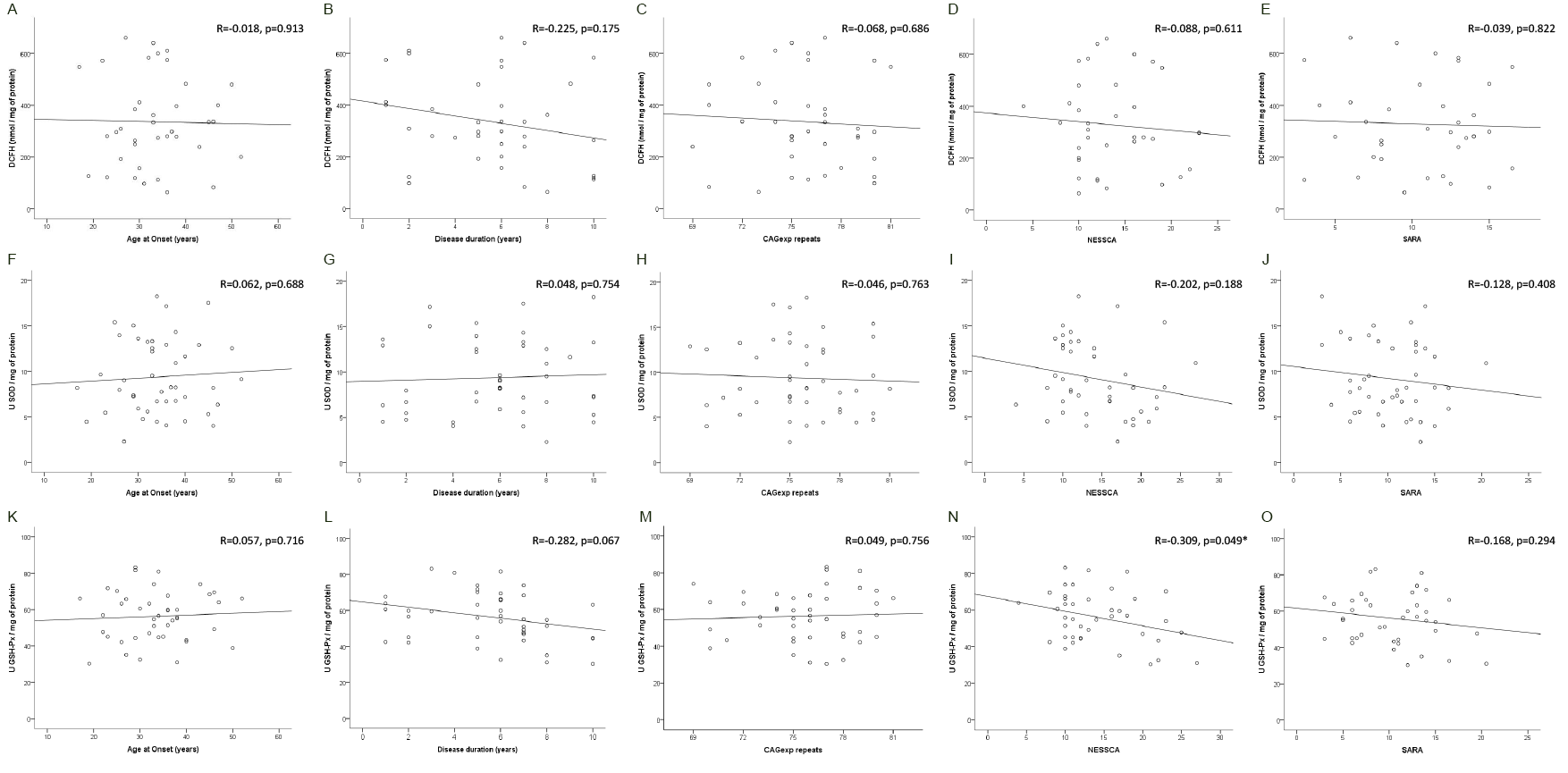

Supplement: Figure S1 — Correlations of redox parameters with clinical and molecular data in the symptomatic SCA3/MJD group. *p < 0.05. DCFH, 2′,7′-dichlorofluorescein diacetate; GSH-Px, glutathione peroxidase; NESSCA, Neurological Examination Score for Spinocerebellar Ataxias; SARA, Scale for the Assessment and Rating of Ataxia; SCA3/MJD, spinocerebellar ataxia type 3/Machado–Joseph disease; SOD, superoxide dismutase. [file image_1.tif]

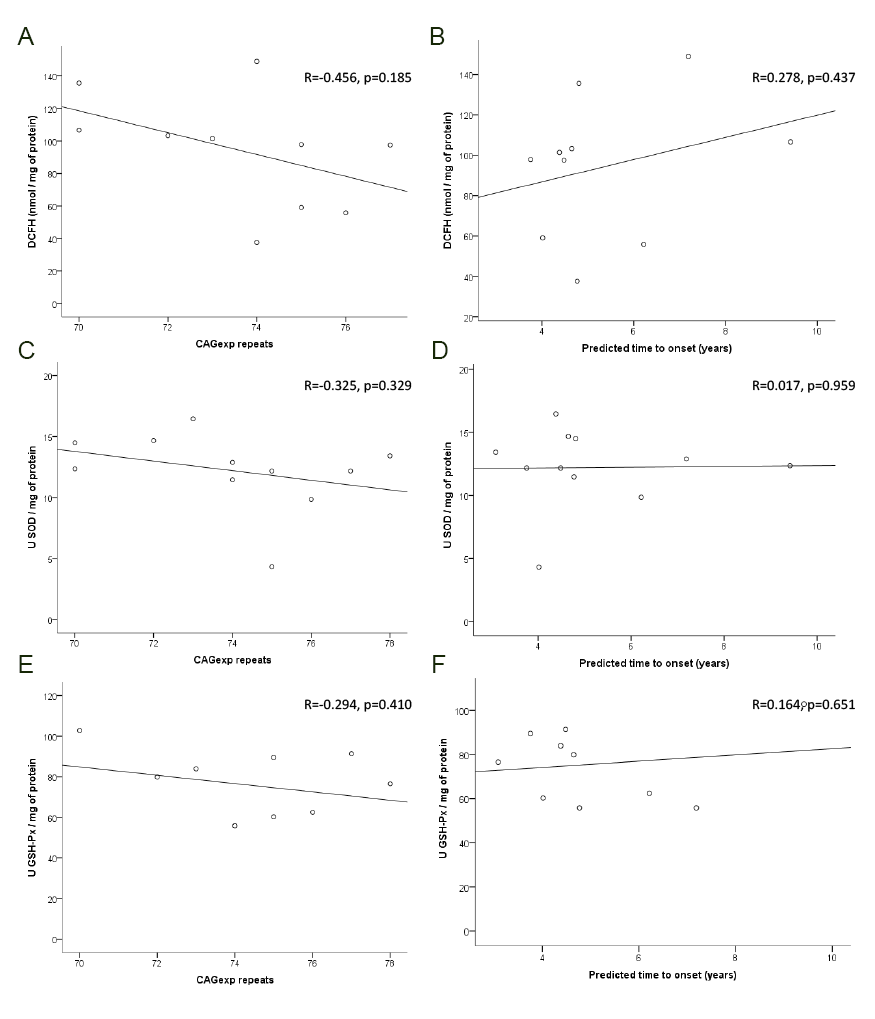

Supplement: Figure S2 — Correlations of redox parameters with clinical and molecular data in the presymptomatic SCA3/MJD group. Predicted time to onset (in years) was calculated with the formula: current age—predicted age of onset [according to Ref. (28)]. DCFH, 2′,7′-dichlorofluorescein diacetate; GSH-Px, glutathione peroxidase; SCA3/MJD, spinocerebellar ataxia type 3/Machado–Joseph disease; SOD, superoxide dismutase. [file image_2.tif]
