# Supplementary material for: Peripheral Oxidative Stress Biomarkers in Spinocerebellar Ataxia Type 3/Machado–Joseph Disease
Source: Front Neurol. 2017 Sep 20;8:485. doi: 10.3389/fneur.2017.00485 (PMC5611390; doi:10.3389/fneur.2017.00485)
Supplement: Supplementary file 3 [file table_1.docx]

**Supplemental Table 1.** Serum levels of oxidative stress markers

|  | Healthy Controls | Symptomatic SCA3/MJD | Presymptomatic |  |
| --- | --- | --- | --- | --- |
|  | Mean (SE) | Mean (SE) | Mean (SE) | *P* |
| DCFH | 182.8 (20.3) | 335.7 (21.2) | 91.8 (42.2) | <0.001^#^ |
| SOD | 10.8 (0.5) | 9.3 (0.5) | 12.3 (1.1) | 0.048^#^ |
| GSH-Px | 70.3 (2.3) | 56.3 (2.4) | 76.8 (5.2) | <0.001^#^ |

Values are given as adjusted means for age. ^#^corrected for age

**Abbreviations:** SE, Standard Error
